# Epidémiologie des accidents domestiques de l’enfant: expérience d’un Service de Pédiatrie Générale du sud tunisien

**DOI:** 10.11604/pamj.2019.33.108.12022

**Published:** 2019-06-12

**Authors:** Ines Maaloul, Sana Kmiha, Sourour Yaich, Kamoun Thouraya, Jamel Damak, Hajer Aloulou, Mongia Hachicha

**Affiliations:** 1 Service de Pédiatrie Générale, CHU Hédi Chaker, Sfax, Tunisie; 2 Service de Médecine Communautaire, CHU Hédi Chaker, Sfax, Tunisie

**Keywords:** Enfant, accident domestique, incidence, intoxication, corps étranger, Child, home accident, incidence, intoxication, foreign body

## Abstract

Les accidents domestiques représentent un problème sérieux de santé publique en pédiatrie. Ils sont responsables d’une lourde morbidité et mortalité parmi la population pédiatrique. Nous avons mené une étude rétrospective colligeant 231 cas d’accidents domestiques chez l’enfant au Service de Pédiatrie de l’Hôpital Hédi Chaker de Sfax durant une période de 5 ans (2008 - 2012). Durant la période d’étude, nous avons colligé 231 cas d’accidents domestiques. Il s’agissait de 124 garçons (53,7%) et 107 filles (46,3%). L’âge moyen était de 2 ans avec des extrêmes de 1 jour et 14 ans. Les enfants âgés de moins de 4 ans étaient les plus exposés (88,7%). Les intoxications accidentelles étaient les accidents les plus fréquents (105 cas). Les caustiques étaient les agents toxiques les plus fréquents (33 cas), suivis par les médicaments (28 cas) puis les hydrocarbures (16 cas). Les accidents par corps étrangers ont représenté le deuxième mécanisme accidentel (64 cas). Il s’agissait de 43 cas d’inhalation de corps étrangers et 21 cas d’ingestion de corps étranger. Nous avons enregistré 28 cas de traumatismes; il s’agissait d’une chute d’une certaine hauteur dans 25 cas. Nous avons recensé 26 cas d’envenimations scorpioniques, 5 noyades, 2 cas de brulure et un seul cas d’électrisation. Les intoxications accidentelles et les accidents par corps étrangers représentent les principaux accidents domestiques dans notre série et la tranche d’âge entre 1 et 4 ans est la plus exposée aux accidents domestiques.

## Abstract (en)

Home accidents are a serious public health problem in Pediatrics. They are responsible for heavy morbidity and mortality in the paediatric population. We conducted a retrospective study of 231 cases of domestic accidents in childhood in the Division of General Pediatrics at the Hedi Chaker Hospital, Sfax over a period of 5 years (2008-2012). During the study period, we collected data from 231 domestic accidents. The study involved 124 boys (53.7 %) and 107 girls (46.3%). The average age of patients was 2 years, ranging from 1 day to 14 years; children under 4 years were the most exposed to home accidents (88.7%). Accidental poisonings were the most common accidents (105 cases). Caustics were the most common toxic agents (33 cases), followed by drugs (28 cases) and hydrocarbons (16 cases). Foreign body accidents were the second most common mechanism of injury (64 cases). They included 43 cases of inhalation of foreign bodies and 21 cases of foreign body ingestion. We recorded 28 cases of trauma, 25 cases were caused by a fall from a certain height. We noted 26 cases of scorpion envenomation, 5 cases of drowning, 2 cases of burn and a single case of electric shock. Accidental poisonings and foreign body accidents were the main home accidents noted during our study and the age group 1 -4 years was the most exposed to home accidents.

## Introduction

Les accidents domestiques (AD) se définissent comme des événements fortuits, dommageables, survenant brutalement au domicile ou ses proches environs [1]. Ils constituent un problème de santé publique majeure dans le monde entier. L’OMS (Organisation Mondiale de la Santé) rapporte que le nombre d’accidents en chiffre absolu est aussi important dans les pays en développement que dans les pays industrialisés. Néanmoins, il est fort probable que la mortalité et le handicap résultant de ces accidents soient plus importants dans les pays en développement [1]. En Tunisie, l’ampleur des AD de l’enfant est méconnue vu l’absence des enquêtes épidémiologiques. L’objectif de ce travail était de déterminer la prévalence des AD parmi la population pédiatrique et de préciser les différents accidents domestiques enregistrés dans un service de pédiatrie générale du sud tunisien.

## Méthodes

Il s’agit d’une étude descriptive, rétrospective, portant sur 231 dossiers durant une période de 5 ans. Ont été inclus tous les enfants âgés de 0 à 14 ans et admis pour accident survenant au domicile ou ses abords immédiats (garage, jardin…). Pour tous les enfants inclus, les données ont été recueillies à partir des dossiers d’hospitalisations dans des fiches préétablies. Les enfants victimes d’un AD, qui n’ont pas nécessité une hospitalisation et les enfants qui ont nécessité d’emblée une hospitalisation en milieu de réanimation ont été exclus. Les données suivantes ont été recueillies: l’âge, le sexe, le niveau socio-économique des parents qui a été évalué à partir des données suivantes: travail des 2 parents et leurs revenus et les conditions du logement, le type de l’accident et l’évolution avec des renseignements spécifiques (brûlure: degré, étendue, agent causal; corps étranger: nature, siège; intoxication: type de toxique, résultat de la fibroscopie digestive haute; chute: lieu et hauteur).

## Résultats

### Aspects épidémiologiques et socio-démographiques

Durant la période d’étude, la prévalence des AD était de 1,37 cas pour 1000 hospitalisations. Les enfants se répartissaient en 124 garçons (53,7 %) et 107 filles (46,3%). L’âge moyen était de 2 ans avec des extrêmes de 1 jour et 14 ans. Les enfants âgés de moins de 4 ans étaient les plus exposés aux AD (88,7%). La fréquence la plus élevée des AD concernait les enfants âgés entre 1 et 4 ans. Les enfants venaient d’une zone urbaine dans 59,3% des cas. Ils étaient issus de familles de bas niveau socio-économique dans 53,1% des cas. L’accident est survenu dans 67,8% des cas au cours de la deuxième moitié de la journée (entre 13 h et 23 H). Les AD étaient enregistrés durant la période estivale dans 34,6% des cas.

### Données spécifiques par type d’accident

**Figure 1:**
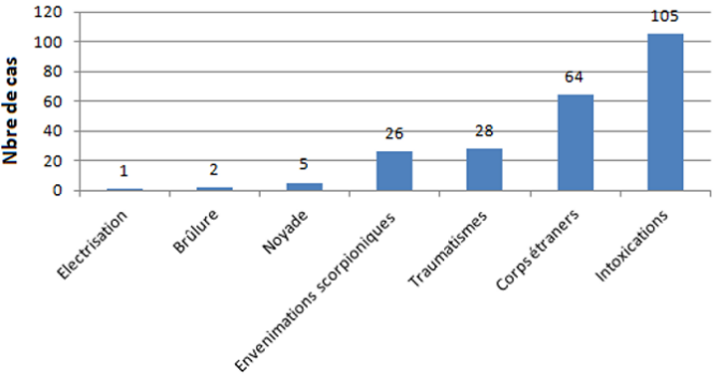
Les différents accidents domestiques retrouvés dans notre série

**Figure 2:**
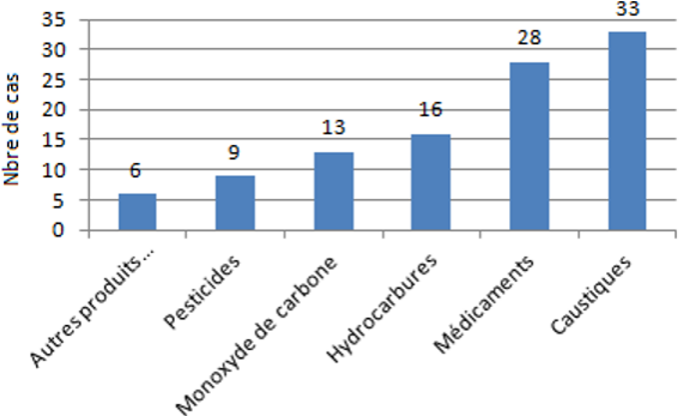
Répartition globale des différents agents toxiques

Les intoxications accidentelles étaient les accidents les plus fréquents (45,5%), suivis par l’ingestion ou inhalation de corps étrangers (27,7%) (Figure 1). Cent-cinq intoxications (45,4%) ont été recensées. Une prédominance masculine a été relevée avec un sex-ratio de 1,5. Les enfants âgés entre 1 et 4 ans étaient les plus atteints (59%). Il s’agissait d’un nourrisson de moins de 1 an dans 27,6% des cas. La victime était responsable directe de son accident dans 80% des cas, une tierce personne était impliquée dans 20% des cas. Les différents agents toxiques retrouvés dans notre série étaient représentées essentiellement par les caustiques (33 cas), suivis par médicaments (28 cas) puis les hydrocarbures (16 cas) (Figure 2). L’eau de javel et la soude étaient les principaux agents caustiques. La fibroscopie digestive haute était faite chez 29 patients, dans un délai moyen de 24 heures suivant l’ingestion de caustique et a objectivé une œsophagite caustique chez 10 patients dont 3 sévères (≥ stade IIb). La soude était l’agent le plus agressif, elle était responsable à elle seule de 7 œsophagites caustiques parmi 10 dont une s’est compliquée de sténose œsophagienne. Les médicaments représentaient le deuxième agent toxique après les produits caustiques (28 cas). Les nourrissons âgés de moins de 1 an étaient les plus touchés (60,7%). Les ocytociques étaient responsables de 32,1% des intoxications médicamenteuses: il s’agissait de nouveaux-nés auxquels les parents avaient administré le *Méthergin^®^* à la place du *stérogyl^®^*.

Nous avons recensé 64 cas de corps étrangers aéro-digestifs. Il s’agissait de 43 cas d’inhalation de corps étrangers et 21 cas d’ingestion de corps étrangers. L’âge moyen était de 28 mois avec des extrêmes de 1 mois et 14 ans. Il s’agit d’une pathologie de la petite enfance puisque 71,9% des cas étaient âgés entre 1 et 4 ans. Une prédominance masculine a été notée (sexe ratio de 1,2). En cas d’inhalation de corps étrangers, les corps étrangers organiques étaient de loin les plus fréquents avec en premier lieu les amandes (23,2%), puis les pois chiche (20,9%), cacahuète (16,2%) et les grains de tournesol (16,2%). La bronchoscopie réalisée dans un délai moyen de 24 heures, a permis l’extraction du corps étranger dans 93% des cas. Un nourrisson de 16 mois est décédé suite à l’inhalation de pois chiche dans un tableau de détresse respiratoire sévère. Concernant l’ingestion de corps étrangers, les corps étrangers inorganiques (pièce de monnaie, bouchon de plastique et pièces métalliques) occupaient le premier rang (71,4%). Une intervention endoscopique était nécessaire dans 42,9% des cas. Une élimination spontanée a été notée dans 57,1%. Aucune complication n’a été constatée en cas d’ingestion de corps étrangers.

Les traumatismes constituent le 3^ème^ mécanisme accidentel. Vingt-huit cas de traumatismes ont été recensés (12,1%). Il s’agissait de 15 filles et 13 garçons, d’âge moyen de 15 mois avec des extrêmes de 1 jour et 6 ans. Ces traumatismes concernaient les enfants âgés de moins de 4 ans dans 27 cas. L’enfant était victime d’une chute d’une certaine hauteur dans 25 cas. Le scanner cérébral réalisé chez 26 patients (92,8%), était pathologique dans 5 cas. Les lésions cérébrales étaient à type d’hématome sous dural (3 cas), hémorragie méningée (3 cas) et contusion cérébrale (1 cas). L’envenimation scorpionique a concerné 26 enfants, d’âge moyen de 2 ans (3 jours - 9 ans). 15 patients étaient classés stade 2 et 2 patients étaient classés stade 3. L’évolution était favorable dans tous les cas.

Cinq cas de noyade avaient été enregistrés avec une prévalence de 1 cas par an. Il s’agissait de 3 garçons et 2 filles d’âge moyen de 1 an avec des extrêmes de 9 et 16 mois. Quatre patients avaient présenté un tableau d’œdème aigu du poumon. Le recours à la ventilation artificielle était nécessaire pour un patient. Un seul enfant a gardé des séquelles à type de retard psychomoteur profond secondaire à l’anoxo-ischémie. Deux enfants âgés respectivement de 9 et 15 mois étaient victimes de brûlures thermiques. Une patiente avait des brulures de 2^ème^ degré étendues sur 7% de la surface corporelle (SC) et l’autre avait des brulures de 2 et 3 degré étendues sur 15% de la SC. Sur le plan évolutif, une patiente est décédée, l’autre a gardé des séquelles esthétiques. Une seule patiente âgée de 1 an, était victime d’une électrisation, elle était asymptomatique et l’évolution était favorable.

## Discussion

Les AD chez l’enfant constituent un problème majeur de santé publique à travers le monde [2]. En France, l’institut de veille sanitaire a mis en place une enquête permanente sur les accidents de la vie courante (AcVC), dont les AD représentent plus de la moitié des cas, qui publie un rapport annuel. Selon les estimations de 2012, 56.168 enfants de moins de 15 ans étaient victimes d’AcVC, chez les nourrissons de moins de 1 an, 80% de ces événements se sont survenus à domicile [3]. Dans une étude prospective, menée en 2011, Ben Hamida et al ont rapporté une incidence annuelle de 14,7% chez l’enfant de moins de 3 ans dans une zone urbaine du Grand Tunis [4]. Dans la plupart des études consacrées à la pathologie accidentelle de l’enfant, la tranche d’âge comprise entre 0 et 5 ans était la plus touchée [1, 5, 6]. Dans notre série, les enfants âgés entre 1 et 4 ans étaient les plus concernés par les AD (64,1%). Ces données peuvent s’expliquer par le fait que l’enfant de moins de 5 ans est en plein développement psychomoteur avec la découverte de l’environnement, la conquête de l’autonomie, les explorations et les expériences nouvelles. La prédominance masculine, rapportée dans la plupart des études chez l’enfant [7], a été également retrouvée dans notre série.

Dans notre série, les intoxications, retrouvées dans 45,5%, représentaient le principal type d’AD. Les intoxications occupaient également la 1^ère^ place dans l’étude de Mabiala-Badela et al. au Congo avec une fréquence de 64% [6]. Dans les pays développés, ce sont plutôt les traumatismes qui constituent le premier mécanisme accidentel [8, 9]. Selon Lavaud [2], les traumatismes représentaient 85% des AD chez l’enfant (Tableau 1). Les intoxications aiguës (IA) représentaient un des principaux accidents domestiques de l’enfant [10, 11]. Les toxiques prévalents diffèrent selon les régions et les activités socioculturelles et économiques. Dans notre série, les enfants s’intoxiquaient le plus souvent par des produits ménagers (52,3%) en particulier les produits caustiques qui représentaient 31,4% de l’ensemble des intoxications. L’eau de javel était le caustique le plus souvent impliqué en raison de sa très grande diffusion et de son utilisation régulière. En Afrique noire, les hydrocarbures représentent le principal agent toxique utilisés comme combustibles ou pour l’éclairage, comme le pétrole lampant [11]. Cependant, en France [12], en Angleterre [13], ainsi qu’au Japon [14], ce sont les médicaments qui étaient le plus souvent incriminés.

**Tableau 1:** Répartition des accidents domestiques selon le mécanisme

	Ingestion de toxique	Chute	Corps étrangers	Brulure	Noyade
**Gabon** Ategbo et al [5]	16,1%	46,6%	7,1%	8,9%	-
**Congo** Mabiala et al [6]	64%	9%	10%	7%	-
**Italie** Sarto et al [10]	-	65%	-	-	-
**Tunisie** Ben Hamida et al [4]	1%	78,2%	-	5,7%	-
**Notre série**	45,5%	12,1%	27,7%	0,8%	2,1%

L’accident par CE est un AD fréquent chez l’enfant, il représentait 2 à 11% du total des AD enregistrés dans les différentes séries [5, 6, 15]. La grande majorité des accidents d’inhalation de CE survient entre l’âge d’acquisition d’une préhension manuelle efficace et celui d’une mastication structurée, soit entre 1 et 4 ans. Dans notre série, 76,7% des cas d’inhalation de corps étranger étaient âgés entre 1 et 4 ans. La nature organique des CE des voies aériennes est largement prédominante, représentée surtout par les corps végétaux en particulier ceux oléagineux (noix, noisette, amandes) [16]. Concernant l’ingestion de corps étranger, les pièces de monnaies sont les plus fréquentes [17]. Ceci a été confirmé dans notre étude avec une fréquence d’ingestion de pièce de monnaie de l’ordre de 28,6%. L’élément clé du diagnostic de CE inhalé est la notion de syndrome de pénétration. Commun à toutes les localisations, il correspond à la mise en jeu des réflexes de défense respiratoire que sont le spasme laryngé et la toux d´expulsion, réactionnels au CE. Il peut passer inaperçu, surtout si l’enfant est seul ou s’il est très jeune [18]. Devant tout syndrome de pénétration, une bronchoscopie au tube rigide doit être réalisée. Le délai de prise en charge de CE inhalés variait selon les séries d’une heure à 2 ans [19, 20]. Dans la série de Mnejja *et al.* [18], la durée de rétention du CE était en moyenne de 16,1 jours avec des extrêmes de 1 jour et 241 jours. Dans notre série, malgré la survenue d’un syndrome de pénétration dans 76,7% des cas, le délai de consultation était tardif, supérieur à 72 heures dans plus d’un tiers des cas.

Les traumatismes constituent la première cause de mortalité des sujets jeunes en particulier des enfants, comme elle peut être à l´origine d´handicaps parfois sévères chez les survivants [21]. La chute est le mécanisme le plus fréquent des traumatismes de l’enfant [22]. Un nouvel AD émergent est le traumatisme lié à la chute du téléviseur. En dépit du marché croissant des écrans plats, 60% des téléviseurs sont à tube cathodique de poids plus élevé (36 à 80 kg), ce qui explique la hausse des accidents liés à la chute de téléviseurs [23]. Dans notre étude, les chutes d’un lit représentaient 32,1% de tous les traumatismes et les accidents liés à la chute de téléviseurs représentaient 7,1%.

En Tunisie, Le scorpionisme représente un véritable fléau social dans les deux tiers du territoire tunisien. Nous enregistrons 30.000 piqûres de scorpion par an [24]. Les enfants âgés de moins de 5 ans étaient les plus touchés [24]. En Tunisie, Bahloul *et al.* [25], trouvait que la majorité des patients (56,7%) étaient d’âge inférieur à 5 ans. Dans notre étude, 88,5% des patients étaient d’âge inférieur à 5 ans.

Les noyades accidentelles de l’enfant sont des évènements graves et parfois mortels. Tout nourrisson peut se noyer dans 20cm d’eau à cause de quelques minutes d’inattention. Les séquelles neurologiques d’origine anoxo-ischémique représentaient 5 à 10% selon les séries [26].

Parmi les AD de l’enfant, la brûlure occupe une place très particulière car elle induit, dans de nombreux cas, des séquelles esthétiques et/ou fonctionnelles dont l’incidence psychologique est lourde et retentit longtemps à distance de l’événement [27]. Les brûlures thermiques sont les plus fréquents [27, 28], ce qui concordait avec nos résultats. Les lésions provoquées étaient peu étendues avec une surface corporelle brûlée moyenne allant de 8 à 21% selon les séries [27, 28]. Dans notre étude, la surface corporelle brûlée moyenne était de 11%. Les enfants sont exposés à un risque élevé de décès par suite de brûlures, le taux mondial étant de 3,9 décès pour 100 000 habitants [29]. Ceci est expliqué par le fait que chez le nourrisson, toute brûlure > 10% peut entraîner une hypovolémie sévère si les apports hydro-électrolytiques ne sont pas adaptés. Le taux de mortalité était de 13,2% selon les séries [27, 28].

Les enfants sont les premières victimes des électrisations par courant de bas voltage lors d´accidents domestiques et représentent 36% des électrisés en France. Les électrisations par courant domestique (220 - 240 V) sont secondaires à l’introduction d’un objet conducteur dans une prise de courant ou en y introduisant les doigts ou en touchant ou portant à la bouche un fil dénudé ou une rallonge raccordée au courant ou encore par contact avec un appareil défectueux [30]. Ces circonstances exposent essentiellement à 2 risques: un risque rare de trouble du rythme cardiaque et le risque fréquent de brûlures des extrémités ou de la bouche [30]. Selon des différents auteurs, les électrisations par courant domestique étaient rarement sévères [31, 32].

## Conclusion

Devant le coût élevé surtout en termes de morbidité des AD de l’enfant qui sont des accidents évitables, des mesures de prévention doivent être mises en place. Cette prévention nécessite l’intervention de toutes les structures concernées: la cellule familiale, le personnel médical et paramédical, les industriels et les pouvoirs publics. Pour la prévention primaire, l’éducation des parents est d’une importance capitale, par le biais des méthodes audio-visuelles, des brochures et de la presse spécialisée. Le rôle des pouvoirs publics et des industriels est important en instaurant des programmes de sensibilisation et d’éducation du grand public et en renforçant la sécurité des produits dangereux. La prévention secondaire vise à diminuer les conséquences de l’AD en éduquant la famille à entreprendre les bons réflexes suite à un AD et en impliquant le personnel médical et paramédical avec la mise en place des centres antipoison travaillant 24H sur 24H , d’une part pour renseigner les parents et d’autre part pour aider les médecins dans leur démarche thérapeutique en cas d’intoxication, qui représente le principal AD dans notre série.

### Etat des connaissances actuelles sur le sujet

- Les accidents domestiques (AD) constituent un problème majeur de santé publique à travers le monde. Les AD par traumatisme sont majoritaires dans les pays développés;
- L’ampleur des AD en Tunisie est méconnue, car les études concernant l’épidémiologie des AD sont rares;
- Des moyens de prévention active et passive ont été mis en place en France avec présence de réseaux de surveillance des accidents de la vie courante entre autre les AD.

### Contribution de notre étude à la connaissance

- Etablir un état des lieux des AD en précisant les aspects épidémiologiques, socio-démographiques, le type de l’accident et les aspects évolutifs;
- Les intoxications représentent le principal AD dans cette série;
- A partir de cet « état des lieux », on peut élaborer des moyens de prévention adaptés: « mieux connaitre pour mieux prévenir »; cette prévention est primaire et secondaire; la prévention primaire est basée surtout sur l’éducation et la sensibilisation des parents et la prévention secondaire consiste surtout à éduquer les parents à avoir les bons réflexes suite à une intoxication et à mettre en place des centres anti-poison.

## Conflits des intérêts

Les auteurs ne déclarent aucun conflit d’intérêts.

## References

[1] Rafai M, Mekaoui M, Chouaib N, Bakkali H, Belyamani L, El Koraichi A (2015). Épidémiologie des accidents domestiques graves de l'enfant admis en réanimation pédiatrique polyvalente à l'hôpital d'enfants de Rabat-Maroc. Pan Afr Med J.

[2] Lavaud J (2002). Accidents de l’enfant.EMC-AKOS. Encyclopédie pratique de médecine.

[3] Institut de veille sanitaire (2012). Enquête permanente sur les accidents de la vie courante. Résultats.

[4] Ben Hamida-Nouaili E, Ben Said A, Ouzini F, Bezzine A, Ben hmida A, Marrakchi Z (2011). Epidémiologie des accidents domestiques du jeune enfant à Tunis; impact de la formation des professionnels de la santé sur la qualité de la collecte d’information. Tun Med.

[5] Ategbo S, Minti OS, Koko J, Mengue Mba-Meyo S (2012). Aspects épidémiologiques des accidents domestiques de l’enfant à Libreville (Gabon). Clinics in Mother and Child Health.

[6] Mabiala-Badela J-R, Pandzou N, Moyen G-M (2010). La pathologie accidentelle du nourrisson aux urgences pédiatriques du CHU de Brazaville. Journal de Pédiatrie et de Puériculture.

[7] Ko AS, Imbert P, Diagne I, Seye MN, Gerardini P, Guyon P (2003). Epidémiologie et pronostic des accidents de l’enfant à Dakar, Sénégal. Med Trop (Mars).

[8] Duval C, Lebrun E (1998). Systéme EHLASS: analyse des accidentsq domestiques chez l’enfant. Ann Fr Anesth Reanim.

[9] Sarto F, Roberti S, Renzulli G, Masiero D, veronese M, Simoncello I (2007). Domestic accidents: a study on children attending the emergency department of the city of Padua. Epidemiol Prev.

[10] Mbika-Cardorelle A, Okoko AR, Ibala R, Moyen G (2003). Epidémiologie des accidents de l’enfant au centre hospitalier universitaire de Brazzaville. Arch Pediatr.

[11] Azoumah KD, Djadou KE, Douti K, Nabroulaba KT, Bakondé B, Agbèrè AD (2008). Les intoxications aiguës accidentelles en milieu hospitalier pédiatrique au CHU de Kara (Togo). Arch Pediatr.

[12] Hue V, Coeugnart-Vanhoucke J, Dubos F, Pruvost I, Martinot A (2011). Accidents de la vie courante observés aux urgences: absence ou échec des moyens de prévention ?. Arch Pediatr.

[13] Jepsen F, Ryan M (2005). Poisoning in children. Current Paediatrics.

[14] Yip WL, Ng HW, Tse ML, Lau FL (2011). An epidemiological study of paediatric poisoning in Hong Kong. HK J Paediatr (New Series).

[15] Viot A, Babin E, Bequignon A, Moreau S, Vadillo M, Valdazo A (2002). Corps étrangers intra-bronchiques de l’enfant. Ann Otolaryngol Chir Cervicofac.

[16] Boufersaoui A, Smati L, Benhalla KN, Boukari R, Smail S, Anik K (2013). Foreign body aspiration in children: experience from 2,624 patients. Int J Pediatr Otorhinolaryngol.

[17] Lakhdar-Idrissi M, Hida M (2011). L’ingestion de corps étranger chez l’enfant: à propos de 105 cas. Arch Pediatr.

[18] Mnejja M, Chakroun AM, Bougacha L, Smaoui L, Ben Salah M, Chakroun A (2012). Bronchoscopie pour inhalation de corps étrangers chez l’enfant: à propos de 223 cas. Arch Pediatr.

[19] Xuechang L, Richard E, Swai H (2011). Airway foreign body aspirations in children at Muhimbili national hospital, Dar es Salaam – Tanzania. East and Central African Journal of Surgery.

[20] Narasimhan KL, Chowdhary SK, Suri S, Mahajan JK, Samujh R, Rao KLN (2002). Foreign body airway obstructions in children – Lessons learnt from a prospective audit. J Indian Assoc Pediatr Surg.

[21] Bahloul M, Chelly H, Ben Hmida M, Ben Hamida C, Ksibi H, Kallel H (2004). Prognosis of traumatic head injury in South Tunisia: a multivariate analysis of 437 cases. J Trauma.

[22] Pomerantz WJ, Gittelman MA, Hornung R, Husseinzadeh H (2012). Falls in children birth to 5 years: different mechanisms lead to different injuries. J Trauma Acute Care Surg.

[23] Claudet I (2010). Les nouveaux accidents domestiques. Arch Pediatr.

[24] Hamouda C, Ben Salah N (2010). Envenimations scorpioniques en Tunisie. Med Emergency.

[25] Bahloul M, Chabchoub I, Chaari A, Chtara K, Kallel H, Dammak H (2010). Scorpion envenomation among children: clinical manifestations and outcome (Analysis of 685 Cases). Am J Trop Med Hyg.

[26] Jan MM (2013). Pediatric near-drowning and drowning. Saudi Med J.

[27] Mercier C, Blond MH (1995). Enquête épidémiologique française sur la brûlure de l’enfant de 0 à 5 ans. Arch Pédiatr.

[28] Messaadi A, Bousselmi K, Korbi A, Chebil M, Oueslati S (2004). Etude prospective de l’épidémiologie des brûlures de l’enfant en Tunisie. Annals of Burns and Fire Disasters.

[29] Peden M, Oyegbite K, Ozanne-Smith J, Hyder AA, Branche C, Fazlur Rahman AKM (2008). Rapport mondial sur la prévention des traumatismes chez l’enfant.

[30] Claudet I, Maréchal C, Debuisson C, Salanne S (2010). Risque de trouble du rythme et électrisation par courant domestique. Arch Pediatr.

[31] Hon KL, Tse WL, Cheung HM, Yip ST, Cheung KL, Wong W (2014). A baby with symmetrical hand injuries and rhabdomyolysis following nonfatal electrocution by an unusual mechanism. Burns.

[32] Celik A, Ergun O, Ozok G (2004). Pediatric electrical injuries: a review of 38 consecutive patients. J Pediatr Surg.

